# 

**Published:** 1875-05

**Authors:** 


ESTABLISHED IN 1855.

VOLUME XIII. | MAY—1875. | NUMBER 2.

S.G.O.L. LIBRARY

### ATLANTA

### Medical and Surgical Journal.

MONTHLY: THREE DOLLARS PER ANNUM, IN ADVANCE.

EDITORS:

ROBERT BATTEY, M.D.,

AND

W. F. WESTMORELAND, M.D.

*PAX ET SCIENTIA, SED VERITAS SINE TIMORE.*

ATLANTA, GEORGIA:

*DUNLOP, WYNNE & CO., BOOK AND JOB PRINTERS.*

1875.

